# Physical education teachers’ knowledge of physical activity recommendations for health promotion in children and adolescents

**DOI:** 10.1038/s41598-023-48522-6

**Published:** 2023-12-10

**Authors:** Adilson Marques, Beatriz Iglésias, Gil Ramos, Élvio R. Gouveia, Gérson Ferrari, João Martins, Pål Lagestad

**Affiliations:** 1 CIPER, Faculdade de Motricidade Humana, Universidade de Lisboa, Lisboa, Portugal; 2 ISAMB, Universidade de Lisboa, Lisboa, Portugal; 3 Faculdade de Motricidade Humana, Universidade de Lisboa, Lisboa, Portugal; 4 Department of Physical Education and Sport, University of Madeira, Funchal, Portugal; 5 LARSYS, Interactive Technologies Institute, Funchal, Madeira Portugal; 6 Escuela de Ciencias de la Actividad Física, el Deporte y la Salud, Universidad de Santiago de Chile (USACH), Santiago, Chile; 7 Laboratorio de Rendimiento Humano, Grupo de Estudio en Educación, Actividad Física y Salud (GEEAFyS), Universidad Católica del Maule, Talca, Chile; 8 Faculty of Teacher Education and Arts, Department of Physical Education and Sport Science, Nord University, Levanger, Norway

**Keywords:** Public health, Quality of life

## Abstract

This study aimed to analyse the knowledge of Portuguese physical education (PE) teachers according to the health recommendations for physical activity (PA) for children and adolescents. A total of 764 teachers participated (55.2% men) with a mean age of 48.2 years. Data was collected using answering to an online survey. Teachers were asked about PA’s frequency, duration and intensity to achieve the recommended level PA. Chi-square was applied to analyse the associations. The main finding is that PE teachers have a lack of knowledge of the health recommendations of PA. Specifically, only 7.5% of the PE teachers in our study could identify the PA recommendations correctly. The intensity component in the health recommendation is the one in which most PE teachers demonstrate correct knowledge of (60.5%), with significant differences in gender and teaching level. However, significantly fewer PE teachers knew the frequency component in the health recommendations for PA (25%), with significant differences in education level. For the duration component, only 37.6% of teachers knew the recommendations. In light of our findings, it is a concern that PE teachers generally lack knowledge according to children’s fulfilment of health recommendations for PA.

## Introduction

The health benefits of physical activity (PA) are well known and described^1,2^. In addition to these benefits, PA facilitates the healthy growth and ageing^1,3^. However, despite the health benefits associated with PA, less than 80% of adolescents engaged in PA regularly^4,5^. Considering the health benefits of PA, recommendations have been developed for people to understand the minimum amount of PA needed. These recommendations serve as guidelines for the practice of health-related PA. For young people aged between 5 and 17 years, the World Health Organization (WHO) recommends at least an average of 60 min a day of moderate to vigorous PA^6^. The recommendation reinforces that the PA should be essentially aerobic and include muscle and bone-strengthening activities at least three times a week^6^.

Because children and adolescents spend almost half of their day at school, and PE is a subject where PA and knowledge of PA is important^7^, PE teachers’ knowledge of PA and the health recommendations are important for promoting healthy living habits in children^8,9^. Furthermore, in many countries, promoting the understanding of the importance of PA for health and healthy and active lifestyles is part of PE goals. For example, in Portugal, the purposes of PE are to reinforce the fondness for the practice of regular physical activities and to, deepen the understanding of its importance as a health factor throughout life, and develop physical fitness in the perspective of improving the quality of life, health and well-being. This knowledge is important because it can be the basis for participation in PA since health-related knowledge might be related to health-related behaviours^10^. PE provides structured and regular PA through most parts of childhood and is therefore considered to be an important contributor to students’ health and knowledge of and behaviour related to PA^8,9,11^.

One of the main purposes of PE is to teach this knowledge about the health recommendations to the students^12^. Therefore, PE teachers must have a good knowledge of these theoretical contents, which is one of the prerequisites for being competent, effective, and confident in their intervention^13^. Nonetheless, it has been found that PE teachers^14,15^ and students^16,17^ do not have adequate knowledge about health-related fitness. However, no studies have examined Portuguese PE teachers’ knowledge about the health recommendations of PA for children and adolescents. In Portugal, less than 4% of students at the end of secondary education could correctly identify the health recommendations for PA, which may indicate a deficit in the knowledge transmitted by PE teachers in this matter^17^. The same concerning findings are found among Portuguese college students^18^. Thus, this study aimed to evaluate the knowledge of Portuguese PE teachers about the WHO PA recommendations for children and adolescents considering different sociodemographics (e.g., sex, age and education level) and teaching characteristics (e.g., years of experience and teaching level).

## Methods

### Procedures and participants

This study employed a cross-sectional design using a large sample of Portuguese PE teachers. An online survey was conducted between January and March 2019. All representative associations of PE teachers in Portugal were contacted. We explained the study’s objectives and requested them to disseminate the questionnaire link to all teachers so they could complete it. Thus, the survey was disseminated through the official mailing contact of the PE teachers. The sample selection was not based on random probability but rather on convenience, as it was chosen non-systematic for practical reasons and ease of access, which may introduce potential biases in the results and limit the generalizability of the findings. This process resulted in a sample of 764 Portuguese PE teachers (55.2% male) with a mean age of 48.2 years (95% confidence interval [CI] = 47.7, 48.7), teaching at the middle and high school levels. In Portugal, there were 8306 PE teachers at the time of data collection. Thus, the maximum margin of error associated with a random sample of 764 respondents was 2.8%, with a confidence level of 95%. Before collecting the data, the study protocol was approved by the Faculty of Human Kinetics, the University of Lisbon’s ethics committee (no. 19/2017), and the Portuguese National Commission for Data Protection (no. 9249/2017). Before completing the survey, teachers informed consent was collected. The study was conducted according to ethical standards in sport and exercise science research^19^.

### Measures

The survey included closed-ended questions regarding sociodemographic data, teaching and PA activity recommendations. Sociodemographic data included sex, age and education level (bachelor, master or doctoral). Years of experience and teaching level (middle school, high school or both) were recorded. Knowledge of the PA recommendations was assessed by a series of three questions assessing each of the recommendations’ components, frequency ‘how many days should children and adolescents engage in PA?’; duration ‘how much time (minimum) in each day should children and adolescents engage in PA?’; and intensity ‘what intensity level (at least) should children and adolescents attain during PA?’. From these three questions, the number of teachers knowing one, two and all components of the PA recommendations were assessed.

### Statistical analysis

Descriptive statistics and 95%CI were calculated for each variable. Chi-square was used to analyse the percentage of teachers knowing each component and knowing one, two or all components of the PA recommendations according to sociodemographic and teaching characteristics. Analysis was performed using the SPSS 28 software (IBM Corp., Armonk, NY, USA). For all analyses, the significance level was set at 0.05.

## Results

Table 1 displays the characteristics of the study participants. Overall, 55.2% were men, 57.6% had ≤ 49 years of age, 49.9% had between 20 and 29 years of experience, 49.6% taught at the middle-school level, and 69% had bachelor’s education level.

**Table 1:** Participants’ characteristics.

	% or mean (95% CI)
Sex	
Male	55.2 (51.7, 58.8)
Female	44.8 (41.2, 48.3)
Age (years)	48.2 (47.7, 48.7)
Age group	
≤ 49 years	57.6 (54.1, 61.1)
≥ 50 years	42.4 (38.9, 45.9)
Years of experience	26.7 (21.2, 32.3)
Years of experience	
≤ 19 years	26.3 (23.2, 29.4)
20–29 years	49.9 (46.3, 53.4)
≥ 30 years	23.8 (20.8, 26.8)
Teaching level	
Middle-school	49.6 (46.1, 53.2)
High-school	12.8 (10.5, 15.2)
Multiple	37.6 (34.1, 41.0)
Education level	
Bachelor	69.0 (65.7, 72.3)
Master	29.5 (26.2, 32.7)
Doctor	1.6 (0.7, 2.5)
Knowing PA recommendations (components)	
Frequency	25.0 (21.9, 28.1)
Duration	37.6 (34.1, 41.0)
Intensity	60.5 (57.0, 63.9)
Knowing PA recommendations	
No	92.5 (90.7, 94.4)
Yes	7.5 (5.6, 9.3)

*CI* Confidence interval; *PA* Physical activity.

Table 2 shows the teachers’ knowledge of the three components of the PA recommendations. The results indicated that only 7.5% (95% CI 5.6, 9.3) of the teachers were aware of all recommendations for PA practice. It is in the frequency component that more teachers were unaware of the PA recommendations (25.0%, 95% CI 21.9, 28.1), and in the intensity component that more teachers demonstrated knowing (60.5%, 95% CI 57.0, 63.9).

**Table 2:** Percentage of teacher’s knowing PA recommendations.

	% (95% CI)
Knowing PA recommendations (components)	
Frequency	25.0 (21.9, 28.1)
Duration	37.6 (34.1, 41.0)
Intensity	60.5 (57.0, 63.9)
Knowing all PA recommendations	
No	92.5 (90.7, 94.4)
Yes	7.5 (5.6, 9.3)

*CI* Confidence interval; *PA* Physical activity.

Table 3 shows the teachers’ knowledge about PA recommendations according to sex, age, years of experience, teaching level, and education level. The Chi-Square test results, referring to the frequency component, revealed significant differences (*p* < 0.001) according to the teacher’s education level. The percentage of teachers with a bachelor’s degree that reported correctly the frequency component was 21.4% (95% CI 17.9, 24.9), while for teachers with a master’s or doctoral degree was 32.9% (95% CI 26.9, 38.9). Thus, teachers with a higher academic degree presented significantly better knowledge about the frequency component of the recommendations.

**Table 3:** Percentage of teacher’s knowledge of each component of the physical activity recommendations by teachers’ characteristics.

	% (95% CI) of teachers knowing PA recommendations (components)					
	Frequency	*p*	Duration	*p*	Intensity	*p*
Sex		0.556		0.155		0.012
Male	24.2 (20.1, 28.3)		39.8 (35.1, 44.5)		64.5 (59.9, 69.0)	
Female	26.0 (21.4, 30.7)		34.8 (29.7, 39.8)		55.6 (50.3, 60.8)	
Age group		0.756		0.077		0.237
≤ 49 years	26.6 (22.5, 30.7)		40.2 (35.6, 44.8)		60.0 (55.4, 64.6)	
≥ 50 years	22.8 (18.3, 27.4)		34.0 (28.8, 39.1)		61.1 (55.8, 66.4)	
Years of experience		0.887		0.007		0.986
≤ 19 years	25.4 (19.4, 31.4)		33.3 (26.8, 39.9)		60.2 (53.4, 67.0)	
20–29 years	25.5 (21.1, 29.8)		43.0 (38.1, 48.0)		60.4 (55.5, 65.3)	
≥ 30 years	23.6 (17.5, 29.8)		30.8 (24.1, 37.5)		61.0 (53.9, 68.1)	
Teaching level		0.507		0.844		0.011
Middle school	26.6 (22.2, 31.1)		38.5 (33.6, 43.4)		56.2 (51.2, 61.2)	
High school	21.4 (13.3, 29.6)		35.7 (26.2, 45.2)		72.4 (63.6, 81.3)	
Multiple levels	24.0 (19.1, 29.0)		36.9 (31.4, 42.5)		62.0 (56.4, 67.6)	
Education level		< 0.001		0.335		0.165
Bachelor	21.4 (17.9, 24.9)		36.4 (32.3, 40.5)		58.8 (54.6, 63.0)	
Master or doctor	32.9 (26.9, 38.9)		40.1 (33.8, 46.3)		64.1 (58.0, 70.2)	

*PA* Physical activity.

In the intensity component, female teachers had significantly lower knowledge than male teachers (female: 55.6%, 95% CI 50.3, 60.8; male: 64.5%, 95% CI 59.9, 69.0, *p* = 0.012). Furthermore, still in the intensity component, the results presented also revealed that teachers of middle school had significantly (*p* = 0.011) lower knowledge (56.2%, 95% CI 51.2, 61.2) than those teaching in high school (72.4%, 95% CI 63.6, 81.3).

Regarding the duration component, there were significant differences (*p* = 0.007) in teachers with 20 to 29 years of experience compared to teachers with more than 30 years of experience, demonstrating the latter more inadequate knowledge of this recommendation’s component (30.8%, 95% CI 24.1, 37.5).

Table 4 presents the general results of PA recommendations knowledge according to teachers’ characteristics. In the level of not knowing any component, the results were significantly different (*p* = 0.021) between teachers with a bachelor’s degree and teachers with a master’s or doctoral degree. It was observed that 24.7% (95% CI 21.0, 28.3) of teachers with a bachelor’s degree were unaware of the recommendations of all components. Regarding this level, the results also indicated that there were no significant differences between sex (*p* = 0.021), age (*p* = 0.570), years of experience (*p* = 0.673), and teaching level (*p* = 0.712).

**Table 4:** Percentage of teachers knowing none, one, two or all components of the physical activity recommendations by teachers’ characteristics.

	% (95% CI) of teachers				
	Not knowing any component	Knowing one component	Knowing two components	Knowing all components	*p*
Sex					0.211
Male	19.2 (15.4, 23.0)	41.7 (37.0, 46.4)	30.6 (26.2, 35.0)	8.5 (5.9, 11.2)	
Female	25.4 (20.8, 30.1)	38.9 (33.7, 44.1)	29.5 (24.7, 34.4)	6.1 (3.6, 8.7)	
Age group					0.57
≤ 49 years	20.5 (16.7, 24.2)	40.2 (35.6, 44.8)	31.4 (27.0, 35.7)	8.0 (5.4, 10.5)	
≥ 50 years	24.1 (19.4, 28.7)	40.7 (35.4, 46.1)	28.4 (23.5, 33.3)	6.8 (4.1, 9.5)	
Years of experience					0.673
≤ 19 years	22.4 (16.6, 28.2)	42.8 (35.9, 49.6)	28.4 (22.1, 34.6)	6.5 (3.1, 9.9)	
20–29 years	20.2 (16.2, 24.2)	39.4 (34.5, 44.3)	31.8 (27.1, 36.4)	8.7 (5.8, 11.5)	
≥ 30 years	25.3 (19.0, 31.6)	40.1 (33.0, 47.2)	28.6 (22.0, 35.1)	6.0 (2.6, 9.5)	
Teaching level					0.712
Middle school	23.0 (18.7, 27.2)	41.2 (36.2, 46.1)	27.4 (22.9, 31.9)	8.4 (5.6, 11.2)	
High school	18.4 (10.7, 26.0)	40.8 (31.1, 50.5)	33.7 (24.3, 43.0)	7.1 (2.0, 12.2)	
Multiple levels	22.0 (17.2, 26.7)	39.4 (33.7, 45.0)	32.4 (27.0, 37.8)	6.3 (3.5, 9.1)	
Education level					0.021
Bachelor	24.7 (21.0, 28.3)	40.2 (36.0, 44.4)	28.8 (25.0, 32.7)	6.3 (4.2, 8.3)	
Master or doctor	16.0 (11.4, 20.7)	40.9 (34.7, 47.2)	32.9 (26.9, 38.9)	10.1 (6.3, 14.0)	

In the three other classes considered (‘Knowing one component’, ‘Knowing two components’, ‘Knowing all components’), no significant differences were observed for any of the discriminated teachers’ groups.

## Discussion

The present study sought to analyse the knowledge of Portuguese PE teachers about the WHO PA recommendations for children and adolescents concerning three components: frequency, duration, and intensity. Based on the results, it was found that most teachers are not fully aware of the PA recommendations, which follow previous works^20^.

PE teachers’ especially important role in contributing to children’s knowledge according to the fulfilment of the health recommendations for PA, combined with their lack of knowledge about the PA recommendations, is somehow problematic, and requires reflection^14,15,21^. Knowledge about PA recommendations is included in health-related exercise (HRE), and previous research shows that teachers also demonstrate little knowledge in this broader area^14,15^. It is necessary to understand the causes of this lack of knowledge about PA and HRE, so that actions can be taken to reverse this trend and optimise student learning through a good knowledge of PE teachers^13,22^.

One of the reasons for this lack of knowledge could be the preconceived beliefs of PE teachers, often established before initial training^23^. It is possible that the teachers who participated in this study had beliefs that PA and HRE were not the main objectives of PE. It can support the discrepancy between different approaches in implementing PE curriculum, i.e., PA for health versus sports performance, reinforced by other studies^24,25^. This may be due to rooted and persistent ideologies strongly attached to sport and fitness, where sports participation equates to health^26^. These ideologies permeate the philosophies of many PE teachers that, together with their past experiences in PE and sports, lead them to privilege PA for performance^26,27^.

PE teachers’ apparent lack of knowledge may also be due to the reduced applicability and relevance of initial training courses^15,21^. Content knowledge is acquired from learning and practice (Ward et al., 2017), so initial training programs must offer opportunities to learn and apply knowledge through practice-based activities. Otherwise, knowledge acquisition remains at an informational level, which is memorised but not applied by the teacher^15^. However, most pre-service teachers have not experienced effective health-related PE (HRPE)^20^. A possible solution to this problem would be to reinforce the PA recommendations content for different age groups and their pedagogical implications in the courses that precede or are associated with PE teacher training^20^. Another solution would be to implement, in these courses, an exercise physiology class exclusively for pre-service PE teachers^28^.

Finally, it is possible that the lack of continuing professional development, especially in this area of HRE^15,21,25,26^, may contribute to PE teachers’ lack of knowledge. Many believe that if teachers are to be professionals, then it is necessary for them to continually engage in professional training and development^26^. However, the reality is quite different, as it has been proven that teachers barely participate in continuing professional development^26^. Usually, teachers in continuing professional development prefer courses associated with PA sports (such as gymnastics and other sports) rather than HRE^20^. The lack of participation in continuing professional development in the HRE area is worrying, as this is one of the main tools that can reverse teachers’ lack of knowledge^26^. Perhaps an investment in more dynamic and easily accessible resources, such as informative pamphlets, newsletters, and interactive learning objects, such as explanatory videos, will make it possible to update knowledge in PA and HRE.

Regarding the education level, significant differences were found. Teachers with master’s or doctoral degrees have greater knowledge than teachers with bachelor’s degrees^14,29^. This may lead us to consider that teachers with more training consolidate their learning better and, as such, are more apt to identify the desired knowledge. Considering teachers’ gender, the results partly agree with the literature^14,29^, in which women tend to have less knowledge in PE and HRE. This may suggest that men are more interested or sensitive to acquiring knowledge about PA recommendations, or even have a different view of their importance for professional fulfilment, especially PA intensity.

Future research assessing the comprehensive knowledge of HRE of Portuguese PE teachers is recommended to test further our research outcomes, which focused only on PA recommendations for children and young people. Experimental studies should also be carried out to implement concrete actions that aim to increase knowledge of HRE, such as those discussed in this work either at an initial training courses or continuous development courses, and to monitor changes in teachers' performance and knowledge, selecting the ones that promote greater success. This could improve teachers’ knowledge and skills in HRE needed to boost the quality of teaching.Action-research projects involving the PE teachers, their students, and the other school community members (e.g. teachers from other subjects, families, and school personnel) around the knowledge, significance and practical tips for meeting the PA recommendations can also be developed by taking into account the whole school physical activity approach. For example, the EUMOVE project’s workshops and resources allow all teachers and the school community to know the PA recommendations and have adequate resources to promote and monitor the achievement of the PA recommendations and those related to sedentary behaviour, sleep, and nutrition (https://eumoveproject.eu/). Since the more recent recommendations for children and adolescents aged 15–17 address PA and sedentary behaviour, the last should also be addressed in PE teachers’ courses.

### Study limitations and strengths

There are limitations in this study that must be considered. Although we have a large sample, the survey was sent to a certain group of teachers, which did not assure a random sample. Secondly, the online survey does not allow for a representative sample of all Portuguese teachers but only those with online access. In addition, it allows the consultation of information through other means during the survey. It may also reduce the motivation and seriousness of the participants compared to a face-to-face context. Finally, structuring the questions in multiple-choice makes it possible to answer randomly correctly. Future studies may also consider open-response questions or interviews and quality analyses. Furthermore, the article is based upon a somehow plain research design. However, we will argue that according to the examination of our research question, this strategy created a high validity and reliability. Future research should consider not only the declarative knowledge of PE teachers but also the pedagogical content knowledge on how to effectively use this knowledge to promote students’ learning towards knowing the PA recommendations and to design and implement a set the strategies and actions to reach them. Despite these limitations, some strengths must be underlined. This is the first study to examine Portuguese PE teachers’ knowledge about the health recommendations of PA for children and adolescents. The sample size (764 respondents), which is almost 10% of Portuguese PE teachers, is a merit of this research. Furthermore, various components of PA recommendations (duration, intensity, and frequency) were questioned, which facilitated data analysis and, consequently, the opportunity to draw more detailed conclusions about PE teachers’ knowledge of the health recommendations of PA for children and adolescents. The analysis of the PE teachers characteristics associated with knowing or not knowing the PA recommendations (sex, educational level) is also important to identify specific populations for targeted strategies.

## Conclusion

Despite the importance of PE teachers in promoting healthy lifestyle habits in children, only 7.5% of the PE teachers in our study were aware of the health recommendations for practising PA in children and adolescents. Our study shows a considerable lack of knowledge according to the frequency and the duration component in the health recommendations for PA. In light of these findings, we suggest that more attention must be given to initial and continuous training courses to increase this knowledge among PE teachers, considering their important role in contributing to children’s knowledge according to the fulfilment of the health recommendations for PA. In doing so, the components of the PA recommendations should be addressed, particularly the frequency and duration. Since the healthy behaviours seem to interact and are associated with the PE participation, the sedentary behaviour and sleep recommendations should also be addressed near the PE teachers. The participation of teachers in continuing professional development should also be encouraged, either through the education of regulatory institutions, through the provision of easily accessible and apprehensible materials, or in action research projects where the school and universities can interact. This way, it will be possible to update teachers’ philosophies and knowledge, ultimately contributing to students’ effective passing of knowledge. These strategies will enhance students learning, integral development, acquisition, and implementation of active and long-lasting healthy lifestyles.

### Supplementary Information

Supplementary Information.

## Data Availability

The data are available from the corresponding author on reasonable request.
