# Supplementary material for: Physical education teachers’ knowledge of physical activity recommendations for health promotion in children and adolescents
Source: Sci Rep. 2023 Dec 10;13:21862. doi: 10.1038/s41598-023-48522-6 (PMC10710445; doi:10.1038/s41598-023-48522-6)
Supplement: Supplementary file 1 — Supplementary Information. [file 41598_2023_48522_MOESM1_ESM.pdf]

Survey on Physical Education Teachers' Perceptions of Physical Fitness Tests

With this survey, we aim to understand teachers' perceptions regarding physical fitness tests in Physical Education. The survey is anonymous and confidential. We appreciate your participation and kindly request that you respond to all questions honestly. There are no right or wrong answers.

* Indicates required question

1. I agree to participate in filling out the survey on teachers' perceptions of physical fitness tests. *
   Mark only one option.
   a) Yes.

2. Gender: *
   Mark only one option.
   a) Male.
   b) Female.
   c) Prefer not to say.

3. Age (in years): * ___________

4. Work experience (in years): * ___________

5. Teaching subject group: *
   Mark only one option.
   a) 110.
   b) 260.
   c) 620.

6. Grade level taught: *
   Tick all that apply.
   a) Primary school.
   b) Middle-school level.
   c) High-school level.
   d) Education and training courses.
   e) Professional courses.

7. Academic degree: *
   Mark only one option.
   a) Bachelor's degree.
   b) Master's degree.
   c) Ph.D.

Physical fitness assessment

8. Have you ever received training on the administration of physical fitness tests in Physical Education? *
   Mark only one option.
   a) Yes, I attended the FITescola Training Workshop (50 hours - The FITescola Platform as a support tool for the development of students' physical fitness in Physical Education and School Sports).
   b) Yes, I attended the FITescola Training Workshop (30 hours - Physical Fitness, Academic Success, Health, and Sports Performance - FITescola Platform).
   c) Yes, I attended the Short-Term Course (6 hours - FITescola M3 = More exercise, Greater success, Better future - Physical Fitness in the School Context).
   d) Yes, I attended another course unrelated to FITescola.
   e) No.

8.1. In which academic year did you attend any of the FITescola training sessions mentioned in the previous question? *
Mark only one option.
a) 2015/2016.
b) 2016/2017.
c) 2017/2018.
d) 2018/2019.
e) I did not attend any FITescola training.

9. Do you usually assess the physical fitness of your students? *
Mark only one option.
a) Yes.
b) No.

10. Do you usually share the results of physical fitness tests with your students? *
Mark only one option.
a) Yes.
b) No.

11. Do you consider it important to assess the physical fitness of your students? *
Mark only one option.
a) Yes.
b) No.
11.1. Why? *
____
____
____

12. Do you usually discuss in your classes the importance of physical activity and the development of physical fitness for health? *
Mark only one option.
a) Yes.
b) No.
12.1. Why? *
____
____
____

13. For the promotion of health, what amount of physical activity do you consider should be recommended to children and adolescents? *
13.1. Frequency: *
Mark only one option.
a) 1 time per week.
b) 2 times per week.
c) 3 times per week.
d) 4 times per week.
e) 5 times per week.
f) 6 times per week.
g) Everyday.

13.2. Duration: *
Mark only one option.

a) At least 30 minutes per session.
b) At least 45 minutes per session.
c) At least 60 minutes per session.
d) At least 75 minutes per session.
e) At least 90 minutes per session.

13.3. Intensity: *
Mark only one option.
a) Light.
b) Moderate.
c) Moderate-to-vigorous.
d) Vigorous.

FITescola
FITescola is an online platform for the dissemination of data related to physical fitness, physical activity, and sedentary behaviors, aimed at educating and motivating students for a better quality of life.

14. Do you know FITescola? *
Mark only one option.
a) Yes.
b) No.

15. Do you usually talk about the FITescola platform in your classes? *
Mark only one option.
a) Yes.
b) No.

16. Do you consider FITescola is an effective tool for disseminating data on the physical fitness of young people? *
Mark only one option.
a) Yes.
b) No.

17. What usefulness do you think the FITescola platform can have for Physical Education teachers? *
____
____

18. Do you believe that FITescola can contribute to the promotion of active and healthy lifestyles in young people? *
Mark only one option.
a) Yes.
b) No.

19. Do you consider that it would be important to assess the motor skills of students in the Primary School? *
Mark only one option.
a) Yes.
b) No.
19.1. Why? *
____
____
